# Pure single-mode Rayleigh-Taylor instability for arbitrary Atwood numbers

**DOI:** 10.1038/s41598-020-60207-y

**Published:** 2020-03-06

**Authors:** Wanhai Liu, Xiang Wang, Xingxia Liu, Changping Yu, Ming Fang, Wenhua Ye

**Affiliations:** 1 School of Electronic Information and Electrical Engineering, Tianshui Normal University, Tianshui, 741000 China; 2 Research Center of Computational Physics, Mianyang Normal University, Mianyang, 621000 China; 3 School of Bailie Mechanical Engineering, Lanzhou City University, Lanzhou, 730070 China; 4 LHD, Institute of Mechanics, Chinese Academy of Sciences, Beijing, 100190 China; 5 School of Engineering Science, University of Chinese Academy of Sciences, Beijing, 100049 China; 6 Hypervelocity Institute Aerodynamics, Chinese Aerodynamics, Mianyang, 621000 China

**Keywords:** Applied physics, Fluid dynamics, Plasma physics, Physics

## Abstract

The validity of theoretical investigation on Rayleigh-Taylor instability (RTI) with nonlinearity is quite important, especially for the simplest and the commonest case of a pure single-mode RTI, while its previous explicit solution in weakly nonlinear scheme is found to have several defections. In this paper, this RTI is strictly solved by the method of the potential functions up to the third order at the weakly nonlinear stage for arbitrary Atwood numbers. It is found that the potential solution includes terms of both the stimulating and inhibiting RTI, while the terms of the decreasing RTI are omitted in the classical solution of the weakly nonlinear scheme, resulting in a big difference between these two results. For the pure single-mode cosine perturbation, comparisons among the classical result, the present potential result and numerical simulations, in which the two dimensional Euler equations are used, are carefully performed. Our result is in a better agreement with the numerical simulations than the classical one before the saturation time. To avoid the tedious expressions and improve a larger valid range of the solution, the method of the Taylor expansion is employed and the velocities of the bubble and spike are, respectively, obtained. Comparisons between the improved and the simulation results show that the improved theory can better predict the evolution of the interface from the linear to weakly nonlinear, even to later of the nonlinear stages.

## Introduction

Rayleigh-Taylor instability (RTI)^1,2^ occurs at an interface when a heavier density fluid is overlapping a lighter one in a gravitational field or when the former is pushed by the latter. The RTI plays a leading role in several areas including, but not limited to, astrophysics^3–5^ and inertial confinement fusion (ICF)^6–21^. Due to the importance of the RTI, the relevant investigation on theory, numerical simulation and experiment becomes an interesting field.

For a simple case of a single cosine-mode perturbation at the interface between the heavier fluid overlapping the lighter one, i.e.,

1 $$y={\eta }^{classical}(x,t=0)=\varepsilon \,\cos (kx),$$

with *ε* the amplitude, *k* = 2*π*∕*λ* wave number, *λ* the wavelength of the initial perturbation and *ε* ≪ *λ*, this interface is prone to RTI. This interface initially develops with time at growth rate $\gamma =\sqrt{Agk}$, where *A* = (*ρ*^*h*^ − *ρ*^*l*^)∕(*ρ*^*h*^ + *ρ*^*l*^) is Atwood number, *g* is gravitational acceleration and *ρ*^*h*^ (*ρ*^*l*^) is density of the heavier (lighter) fluid. That is, the perturbed interface evolves in the form

2 $${\eta }^{classical}(x,t)={\eta }_{L}\cos (kx),$$

in which ${\eta }_{L}=\varepsilon \,\exp (\gamma t)$ is called as the linear amplitude of the interface. This is known as the linear theory, see both the Rayleigh and Taylor papers^1,2^. After a short time, this evolution interface includes the second, third harmonics, and so on, because of the mode-coupling effect in a nonlinear system. The appearance of higher harmonics results in the different amplitude growth of the interface in the heavier and lighter fluids. The portion of the interface entering the heavier (lighter) fluid is named as a bubble (spike). Generally, the spike moves faster than the bubble, especially for the larger Atwood number. The higher harmonics play an important role for the amplitude difference between the bubble and spike. The evolution amplitudes of the first three harmonics are given in weakly nonlinear theory^22–25^, which are as below:

3a $${\eta }_{1}^{classical}={\eta }_{1,1}^{classical}+{\eta }_{3,1}^{classical}={\eta }_{L}-\frac{1}{16}\left(3{A}^{2}+1\right){k}^{2}{{\eta }_{L}}^{3},$$

3b $${\eta }_{2}^{classical}=-\frac{1}{2}Ak{{\eta }_{L}}^{2},$$

3c $${\eta }_{3}^{classical}=\frac{1}{2}\left({A}^{2}-\frac{1}{4}\right){k}^{2}{{\eta }_{L}}^{3},$$

where the amplitude of the fundamental mode, ${\eta }_{1}^{classical}$, includes the linear one *η*_*L*_ and the third-order feedback/correction. Within the third-order weakly nonlinear framework, the second and the third harmonics have no feedbacks from higher orders. In the recent work^26^, by employing the tracking technology of the mode coupling up to the fifth order, the effect of the mode-coupling branches/channels on harmonic amplitudes in single-mode classical Rayleigh-Taylor instability for arbitrary Atwood numbers in the weakly nonlinear stage is investigated.

Because the bubble (spike) is the structure which moves the fastest to the heavier (lighter) fluid, its evolution is greatly focused on. For the asymptotical behavior, the expansion method of the potential function was first employed by Layzer to bubbles^27^, and then bubbles and spikes^28–30^. Based on the weakly nonlinear solution, the perturbation solutions can lead to a perfect Padé approximation by matching the linear solution and the asymptotic solution^31–33^.

However, when the above theory of the third-order weakly nonlinearity is deeply investigated, deficiencies are found, resulting in the corresponding prediction largely deviating results from the numerical simulations or experiments. Within the third-order weakly nonlinear framework, the interface equation should be

4 $$\eta (x,t)=\mathop{\sum }\limits_{i=1}^{3}{\eta }_{i}(t)\ \cos (ikx).$$

When the amplitudes of the first three harmonics are replaced with the classical ones (3a-c) the two deficiencies are found as below.

(i) the initial velocity of the whole interface is not equal to zero, while the general RTI starts from the initial interface with the rest;
(ii) the interface is not in the form of the pure single-mode perturbation, but multi-mode perturbations, and then the initial interface condition (1) is not strictly satisfied.

When the linear amplitude is carefully taken into account, it should be

5 $${\eta }_{1}^{linear}=\frac{1}{2}\varepsilon \ \exp (\gamma t)+\frac{1}{2}\varepsilon \ \exp (-\gamma t),$$

which was also obtained by Forbes^34^. In Eq. (5), these two terms play a different role on RTI: the former stimulates the RTI, while the latter inhibits the RTI. Generally speaking, just the stimulating term is focused on, while the inhibiting term is neglected. As a result, the prediction of the linear amplitude without the inhibiting term is always higher than that with this term. For a nonlinear system, due to the effect of the nonlinear mode coupling, the neglect of the inhibiting term in the first order can make not only the inhibiting terms but also the stimulating ones disappear. That is, in the weakly nonlinear stage, the disappearance terms including the inhibiting and stimulating ones can make the prediction have a larger difference from the related results of the numerical simulations or experiments. Meanwhile, this classical solution of the weakly nonlinear stage can mislead the readers and the researchers for better understanding the weakly nonlinearity, even the whole nonlinearity. In this paper, the evolution equations of the interface are strictly solved, and the solutions satisfying the condition of the initial velocity, together with the pure single-mode perturbation, are obtained order by order.

Therefore, this work raised the linearized solution, while the previous works neglected the role related to the inhibiting terms ($\exp [-\gamma t]$), resulting to a big difference between the third theory of the weakly nonlinearity and the numerical simulations. Based on the pure single-mode RTI, our work presents the third-order theory of the weakly nonlinearity with the role of the whole terms, and predicts the better results from the initial linear stage to the later stage of the nonlinearity.

## Theory Framework and Explicit Solutions

When two incompressible, inviscid and irrotational fluids with arbitrary density rations (i.e., arbitrary Atwood numbers) in two dimensions are taken into account, the governing equations are

6a $$\bigtriangleup {\phi }^{i}(x,y,t)=0,\ \ \,{\rm{in\; two\; fluids}}\,$$

6b $$\frac{\partial \eta }{\partial t}+\frac{\partial \eta }{\partial x}\frac{\partial {\phi }^{i}}{\partial x}-\frac{\partial {\phi }^{i}}{\partial y}=0\,{\rm{at}}\,y=\eta (x,t),$$

6c $$\begin{array}{ccc} &  & {\rho }^{h}\frac{\partial {\phi }^{h}}{\partial t}-{\rho }^{l}\frac{\partial {\phi }^{l}}{\partial t}+\frac{1}{2}{\rho }^{h}\left[{\left(\frac{\partial {\phi }^{h}}{\partial x}\right)}^{2}+{\left(\frac{\partial {\phi }^{h}}{\partial y}\right)}^{2}\right]\\  &  & \ \ -\frac{1}{2}{\rho }^{l}\left[{\left(\frac{\partial {\phi }^{l}}{\partial x}\right)}^{2}+{\left(\frac{\partial {\phi }^{l}}{\partial y}\right)}^{2}\right]+({\rho }^{h}-{\rho }^{l})g\eta +f(t)=0\,{\rm{at}}\,\ \ y=\eta (x,t),\end{array}$$

where *y* = *η*(*x*, *t*) is the vertical position of the interface at time *t*, *ϕ*^*i*^(*x*, *y*, *t*) is the velocity potential, *f*(*t*) is a function of the arbitrary time which is an integral constant generated in the derivation of the Eq.  (6c) by employing method of the potential flow, and the superscript *i* denotes *h* or *l*. Equation (6a) is Laplace equation, Eqs. (6b) and  (6c) are, respectively, the continuous conditions of the normal velocity and pressure at the evolution interface.

When the heavier fluid is overlapping the lighter one in the gravitational field of acceleration $\overrightarrow{g}=-g{\overrightarrow{e}}_{y}$, any perturbations at the interface can arouse the rest interface. Here, we consider the pure single-mode cosine perturbation in the form of

7 $$\eta (x,t=0)=\varepsilon \ \cos \ (kx),$$

with the initial velocity being

8 $$v(x,t=0)=0$$

According to the mode coupling rule, the evolution interface, within the three-order framework, is given by Eq. ((4)) where the amplitude of the fundamental mode *η*_1_(*t*) = *η*_1,1_(*t*) + *η*_3,1_(*t*). The velocity potentials of the fluids, satisfying their Laplace equations and infinity conditions, can be expressed as

9 $${\phi }^{h}(x,y,t)=[{\phi }_{1,1}^{h}(t)+{\phi }_{3,1}^{h}(t)]{e}^{-ky}\cos (kx)+{\phi }_{2}^{h}(t){e}^{-2ky}\cos (2kx)+{\phi }_{3}^{h}(t){e}^{-3ky}\cos (3kx).$$

10 $${\phi }^{l}(x,y,t)=\left[{\phi }_{1,1}^{l}(t)+{\phi }_{3,1}^{l}(t)\right]{e}^{ky}\cos (kx)+{\phi }_{2}^{l}(t){e}^{2ky}\cos \ (2kx)+{\phi }_{3}^{l}(t){e}^{3ky}\cos \ (3kx).$$

The time coefficients of the evolution interface need to be solved at last. The solving steps are listed as:

(i) substitute Eqs. (4), (9) and (10) to Eqs. (6b) and (6c);
(ii) replace *y* in the new equations with function *η*(*x*, *t*) [see Eq. (4)];
(iii) move the non-zero terms to the left hand side from the right of the each equation;
(iv) expand the expressions into the third-order (*ε*^3^) Taylor series at *ε* = 0;
(v) construct an ordinary differential equation for the first-order, second-order, and third-order terms, respectively, consisting of the coefficients of *ε*, *ε*^2^, and *ε*^3^;
(vi) eliminate the unknown coefficients of the velocity potential, and obtain the corresponding deference equations on amplitude coefficients of the evolution interface.

A second-order ordinary differential equation for the first-order terms together with the initial conditions is given as below:

11a $${\ddot{\eta }}_{1,1}(t)-{\gamma }^{2}{\eta }_{1,1}(t)=0,$$

11b $${\eta }_{1,1}(0)=\varepsilon ,$$

11c $${\dot{\eta }}_{1,1}(0)=0,$$

where the double dots denote the second derivative of the time and the single dot denotes the first derivative of time. The solution is obtained as

12 $${\eta }_{1,1}(t)=\varepsilon \ \cosh (\gamma t).$$

As to differential equations for the second and the third terms, the initial values of the amplitude and velocity, ${\eta }_{2}(0),{\dot{\eta }}_{2}(0),{\eta }_{3,1}(0),{\dot{\eta }}_{3,1}(0),{\eta }_{3}(0),{\dot{\eta }}_{3}(0)$, are zero and the differential equations are below:

13a $${\ddot{\eta }}_{2}(t)-2{\gamma }^{2}{\eta }_{2}(t)+Ak{\gamma }^{2}\ {\sinh }^{2}(t\gamma )=0,$$

13b $${\ddot{\eta }}_{3,1}(t)-{\gamma }^{2}{\eta }_{3,1}(t)+\xi (t)=0,$$

13c $${\ddot{\eta }}_{3}(t)-3{\gamma }^{2}{\eta }_{3}(t)+\zeta (t)=0$$

where

14 $$\xi (t)=\frac{1}{16}{k}^{2}{\gamma }^{2}{e}^{\left(-3-\sqrt{2}\right)t\gamma }\left[{A}^{2}\upsilon +{e}^{\sqrt{2}t\gamma }({e}^{2t\gamma }+1)({e}^{4t\gamma }+1)\right],$$

$$\text{with}\,\upsilon =-2\left(1+\sqrt{2}\right){e}^{2t\gamma }+2\left(\sqrt{2}-1\right){e}^{4t\gamma }+3{e}^{\sqrt{2}t\gamma }+2\left(\sqrt{2}-1\right){e}^{2\left(1+\sqrt{2}\right)t\gamma }+{e}^{\left(2+\sqrt{2}\right)t\gamma }$$$$-2\left(1+\sqrt{2}\right){e}^{2\left(2+\sqrt{2}\right)t\gamma }+{e}^{\left(4+\sqrt{2}\right)t\gamma }+3{e}^{\left(6+\sqrt{2}\right)t\gamma }$$,

and

15 $$\zeta (t)=\frac{3}{32}{k}^{2}{\gamma }^{2}{e}^{\left(-3-\sqrt{2}\right)t\gamma }\left({e}^{2t\gamma }-1\right)\ \left[{e}^{\sqrt{2}t\gamma }\left({e}^{4t\gamma }-1\right)-4{A}^{2}\omega \right],$$

with $$\omega =\sqrt{2}{e}^{2t\gamma }-{e}^{\sqrt{2}t\gamma }-\sqrt{2}{e}^{2\left(1+\sqrt{2}\right)t\gamma }+{e}^{\left(4+\sqrt{2}\right)t\gamma }.$$

The initial conditions are taken into account, these equations can be solved in turn. The amplitudes of the first three harmonics are expressed as bellow.

16a $$\begin{array}{lll}{\eta }_{1}(t) & = & \varepsilon \cosh (\gamma t)+\frac{1}{128}{k}^{2}{\varepsilon }^{3}\left[-8t\gamma \sinh (t\gamma )-4\,\sinh (2t\gamma )\sinh (t\gamma )+\alpha {A}^{2}\right]\\  & = & {\eta }_{1}^{classical}+{\eta }_{1}^{present},\end{array}$$

16b $$\begin{array}{lll}{\eta }_{2}(t) & = & -\frac{1}{4}Ak{\varepsilon }^{2}\left[\cosh (2\gamma t)-2\cosh \left(\sqrt{2}\gamma t\right)+1\right]\\  & = & {\eta }_{2}^{classical}+{\eta }_{2}^{present},\end{array}$$

16c $$\begin{array}{lll}{\eta }_{3}(t) & = & \frac{1}{64}{k}^{2}{\varepsilon }^{3}\beta \\  & = & {\eta }_{3}^{classical}+{\eta }_{3}^{present},\end{array}$$

where

$$\begin{array}{ccc} &  & \alpha =8{e}^{(-1-\sqrt{2})\gamma t}({e}^{2\sqrt{2}\gamma t}+1)+8{e}^{(1-\sqrt{2})\gamma t}({e}^{2\sqrt{2}\gamma t}+1)-8\gamma t\sinh (\gamma t)-26\cosh (\gamma t)-6\cosh (3\gamma t),\\  &  & \beta =(8{A}^{2}-2)\cosh (3\gamma t)+8(2{A}^{2}+1)\cosh (\sqrt{3}\gamma t)-48{A}^{2}\cosh (\gamma t)\cosh (\sqrt{2}\gamma t)\\  &  & +\,6(4{A}^{2}-1)\cosh (\gamma t),\\  &  & {\eta }_{1}^{present}=\varepsilon \left[\cosh (\gamma t)-{e}^{\gamma t}\right]+{k}^{2}{\varepsilon }^{3}\left[\alpha {A}^{2}+8(3{A}^{2}+1){e}^{3\gamma t}-4\sinh (\gamma t)(2\gamma t+\sinh (2\gamma t))\right]/128,\\  &  & {\eta }_{2}^{present}=Ak{\varepsilon }^{2}\left[2{e}^{2\gamma t}-\cosh (2\gamma t)+2\cosh (\sqrt{2}\gamma t)-1\right]/4,\\  &  & {\eta }_{3}^{present}={k}^{2}{\varepsilon }^{3}\left[(8-32{A}^{2}){e}^{3\gamma t}+\beta \right]/64.\end{array}$$

As mentioned above, from the results one finds that there are several terms which either stimulate or inhibit the RTI, especially for the solutions from the third order. These solutions consist of hyperbolic sine and cosine functions including stimulating and inhibiting terms. That is to say, if the inhibiting term in the linear solution is deleted, many terms will disappear.

## Comparison between the Results of the Theory and the Numerical Simulations

In order to validate the present theory, the results of the classical and the present theories are compared with the numerical simulation. The code, based on CFD^2^, used here is developed by our research team. For the two dimensional Euler equations of the inviscid and compressible fluid dynamics, fifth-order finite difference WENO schemes with the third-order Runge-kutta time discretization are used. In our numerical simulations, we set up the problem, in which the related parameters step from the ref. ^35^, as follows: the computational domain is [0, 0.25] × [0, 1]; the pure single-mode cosine perturbation interface between the down lighter fluid of the density *ρ*^*l*^ = 1 and up heavier *ρ*^*h*^ = 1.5 for *A* = 0.2 and *ρ*^*h*^ = 9.0 for *A* = 0.8 is $y=0.5+0.005\ \cos (8\pi x)$ with the acceleration in the negative *y*-direction; the pressure $p={p}_{1}-g{\int }_{1}^{y}\rho (\mu )d\mu$ with the initial pressure *p*_1_ = 1 at *y* = 1 boundary and gravitational acceleration *g* = 1 and it is continuous across the interface. Hence, for the top and bottom boundaries, the pressure *p*_1_ = 1, velocity ${\overrightarrow{v}}_{1}=0$ at *y* = 1 and the pressure *p*_0_ = 2.25 (*p*_0_ = 6) for *A* = 0.2 (*A* = 0.8), velocity ${\overrightarrow{v}}_{0}=0$ at *y* = 0. The periodic boundary conditions are imposed for the left and right boundaries. It should be noted that in the classical RTI, the thickness of the two fluids tends to being infinity, i.e., *y* → ± *∞*, and all the physical quantities keep invariable at *y* → ± *∞* whatever RTI evolves quickly between the two fluids. However, in the numerical simulation, the domain of the computation is always limited. In order to better verifying our provided theory which is governed by Laplace Eq. (6a) together with interface conditions (6b–c), in our numerical simulation governed by Euler equations, as stated just now, the domain of the computation in *y* direction is selected as [0, 1]. When the initial perturbation in *y* = 0.5 starts evolving in the weakly nonlinear scheme, the top and bottom boundaries can not receive any changes. Hence, in the numerical simulation all the physical quantities keep invariable in the boundaries *y* = 0 and *y* = 1.

For the whole field of the fluids, the initial velocity in the *x*- or *y*-direction is zero. Because of the RTI, the interface perturbation will grow with time. The top of the bubble (spike) is located at *x* = 0(*x* = 0.125) and the corresponding amplitude or velocity can be obtained by tracking its location at different time.

For the whole evolution interface in the RTI, the top of the bubble or the spike is the structure which grows fastest and has the longest amplitude. In our explicit interface (4), the tops of the bubble and the spike are located at *x* = 0 and *x* = *π*∕*k*, and their amplitudes are, respectively, *η*_*bb*_(*t*) = *η*(*x* = 0, *t*) and *η*_*sp*_(*t*) = |*η*(*x* = *π*/*k*, *t*)|. Here, the positive amplitudes are taken into our account, and then their velocities are, respectively, ${v}_{bb}(t)=\dot{\eta }(x=0,t)$ and ${v}_{sp}(t)=\dot{\eta }(x=\pi /k,t)$. Due to the tedious expressions of the amplitude and the velocity of the bubble and spike, they are not explicitly given here.

**Figure 1:**
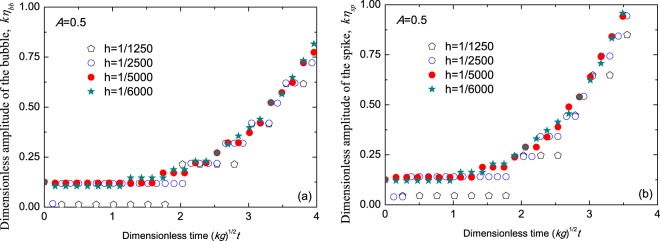
Comparisons of the dimensionless amplitudes of the bubbles (**a**) and the spikes (**b**) among the mesh steps *h* = 1/1250 (pentagons), *h* = 1/2500 (circles), *h* = 1/5000 (solid circles), and *h* = 1/6000 (solid stars) for Atwood number *A* = 0.5 vs dimensionless time $\sqrt{kg}t$. The initial amplitude of the interface is *kε* = 0.1256.

In order to determine the value of the mesh step *h*, some results of the mesh convergence analysis are shown in Fig. 1. From comparisons of the dimensionless amplitudes of the bubbles and the spikes among the mesh steps 1/1250, 1/2500, 1/5000 and 1/6000 in Fig. 1, the mesh step is set as the uniform *h* = 2 × 10^−4^ in our following computations.

**Figure 2:**
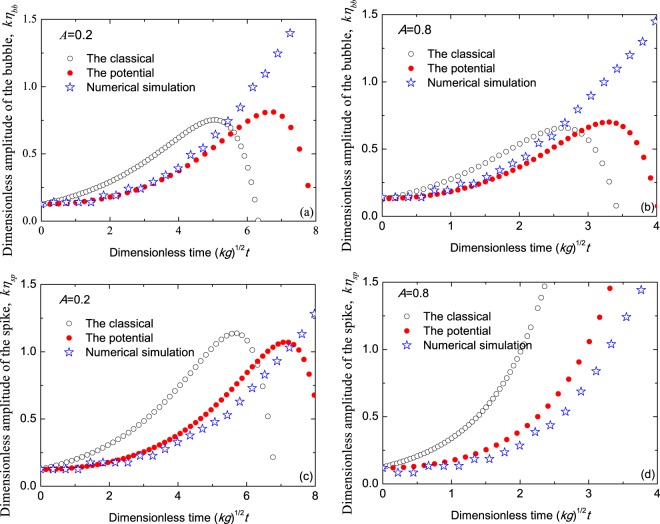
Comparisons of the dimensionless amplitudes of the bubbles (top **a**,**b**) and the spikes (bottom **c** and **d**) among the classical theory (circles), the present potential theory (dots) and the numerical simulation (stars) for Atwood numbers *A* = 0.2 (**a**,**c**) and *A* = 0.8 (**b**,**d**) vs dimensionless time $\sqrt{kg}t$. The initial amplitude of the interface is *kε* = 0.1256.

**Figure 3:**
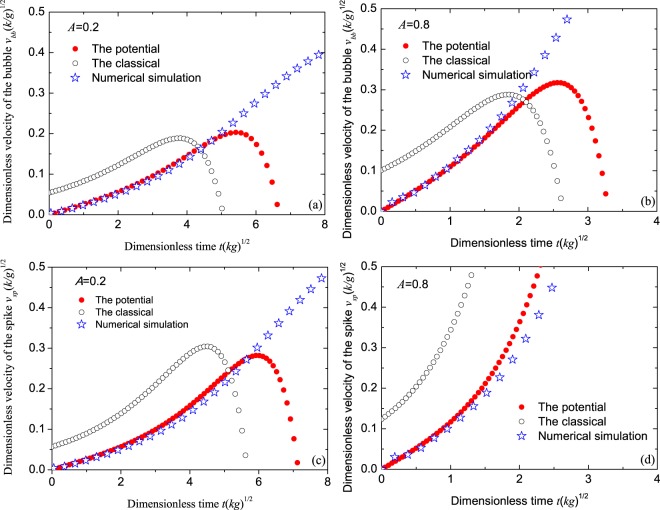
Comparisons of the dimensionless velocities $v\sqrt{k/g}$ of the bubbles top (**a**,**b**) and the spikes (bottom **c,d**) among the classical theory (circles), the present potential theory (dots) and the numerical simulation (stars) for Atwood numbers *A* = 0.2 (**a**,**c**) and *A* = 0.8 (**b**,**d**) vs dimensionless time $\sqrt{kg}t$. The initial amplitude of the interface is *kε* = 0.1256.

The amplitude and velocity of the bubble and spike from the classical theory (denoted by circles), the present potential theory (denoted by dots) and numerical simulation (denoted by stars) are, respectively, compared in Figs. 2 and  3 for Atwood number *A* = 0.2 (left) and *A* = 0.8 (right). Here, the perturbation wave number *k* and gravitational acceleration *g* are used to normalized the time, amplitude and velocity.

From Fig. 2, one finds that in weakly nonlinear stage, the prediction of the present potential theory is in good agreement with the results of the numerical simulation, while the amplitude predicted by the classical theory is always larger than our result. Because of the negative feedback to the fundamental mode from the third order, the amplitude of the the bubble first increases up to its maximum at the saturation time, and then decreases with time for *A* = 0.2 and *A* = 0.8. It is obvious that the saturation time predicted by the classical theory is smaller than ours. That is to say, our theory predicts the range of the valid time longer than the classical. For *A* = 0.8 spike, the feedback to the fundamental mode from the third order is not negative, but positive, resulting in the amplitude of the spike growing all the time. Anyway, it is found that the present potential theory gives a better agreement with the numerical simulation for longer time than the classical.

As stated above, the classical theory did not predict the RTI with the initial velocity being zero, as shown in Fig. 3. It is obvious that the velocity predicted by the classical theory is larger than ours. Our results agree better with the numerical simulations for *A* = 0.2 and *A* = 0.8.

Considering the tedious expressions of the amplitude and the velocity of the bubble and spike, together with the larger range of the valid time in the weakly nonlinear stage predicted by present potential theory, the expressions of the velocity of the bubble and spike are expanded in Taylor series in the dimensionless time $\tau =\sqrt{gk}t$ up to the third order

17a $${\widehat{v}}_{bb}=\frac{1}{4}A\sigma \left(4-{\sigma }^{2}\right)\tau +\frac{1}{6}{A}^{2}\sigma (-2A\sigma -3{\sigma }^{2}+1){\tau }^{3},$$

17b $${\widehat{v}}_{sp}={\widehat{v}}_{bb}+\frac{2}{3}{A}^{3}{\sigma }^{2}{\tau }^{3},$$

with initial amplitude *σ* = *kε* and $\widehat{v}=v\sqrt{k/g}$.

**Figure 4:**
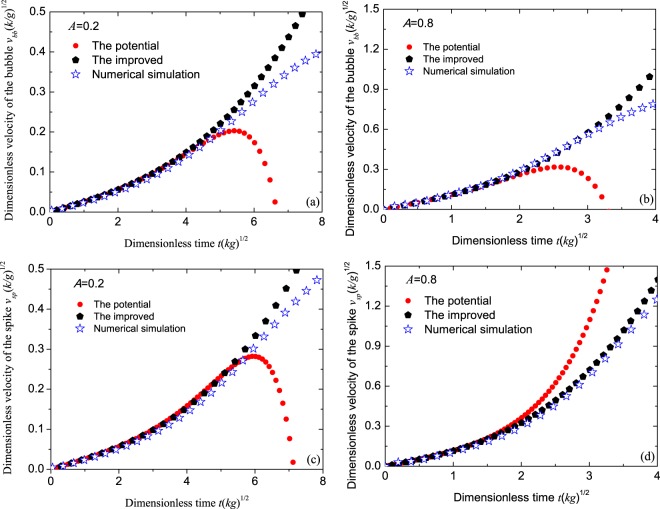
Comparisons of the dimensionless velocities $v\sqrt{k/g}$ of the bubbles (top **a**,**b**) and spikes (bottom **c**,**d**) among the present potential theory (dots), the present improved theory (pentagons) and the numerical simulation (stars) for Atwood numbers *A* = 0.2 (**a**,**c**) and *A* = 0.8 (**b**,**d**) vs dimensionless time $\sqrt{kg}t$. The initial amplitude of the interface is *kε* = 0.1256.

For the sake of checking whether above velocity formulas are effective, the improved velocities predicted by Eqs. (18a) and (18b) (denoted by pentagons), our potential theory (denoted by dots) and the numerical simulations (denoted by stars) are compared in   Fig. 4. It is found that the improved theory predicts a better result than our potential theory. On the one hand, the results of these two theories are in good agreement in the early stage of the weakly nonlinearity. On the other hand, our improved theory extends the valid range of our potential theory, either for the bubble or the spike, especially for the larger Atwood number, as can be affirmed by the good agreement of our potential theory and the numerical simulations in Fig. 4.

In fact, these purely potential-flow solutions only exist for a finite time interval, since they are ultimately killed at a finite time, by the curvature singularity at the interface predicted by Moore^36^. In this paper, the valid time of the improved theory is extended due to two factors. One is the the theory of the weakly nonlinearity containing the whole terms [not only the term $\exp (\gamma t)$ but also the one $\exp (-\gamma t)$]. The role of the inhibiting terms can not be neglected. The other is the application of Taylor expansion. These two aspects play an important role.

## Conclusion

In this paper, we employ the method of the small parameter expansion of the potential function with nonlinear corrections up to the third order to analytically explore the history evolution of the bubble and the spike for the pure single-mode cosine perturbation at the interface between two different fluids in classical (irrotational, incompressible, and inviscid fluids) RTI. First, we analysis the defects in the classical results: (i) the initial interface is not in the form of the pure single mode, but in the multi mode; (2) the initial velocity of the interface perturbation is not zero. These two defects are resulted from the absence of the time inhibiting term in the linear solution. As a result, we keep the inhibiting term in the linear solution, and obtain the solutions satisfying the differential equations of the evolution interface in potential functions. The comparisons of the amplitude and the velocity evolutions of the bubble and the spike are performed among the classical, our potential theories and our numerical simulations. It is found that before the saturation time, the results of the potential theory agree with the numerical simulations better than the classical one. After the saturation time, the results of the classical and our present theories start decreasing rapidly with time due to the negative feedback to the fundamental mode from the third order. For this reason, we expand our potential results of the velocities of the bubble and the spike in Taylor series in time, and obtain the improved theory. Comparisons among the potential theory, the improved theory and numerical simulations show that the improved theory predicts a better result, either for the velocities of the bubble or the spike, and the valid range of the time is also larger than the potential theory, as can be confirmed in Fig. 4.
